# Adaptation and validation of the Berkeley Expressivity Questionnaire among Chinese international students in Malaysian higher education

**DOI:** 10.3389/fpsyg.2025.1600302

**Published:** 2025-05-30

**Authors:** Min Xu, Xiaohan Yang, Hadijah Jaffri, Boon Yew Wong

**Affiliations:** University of Technology Malaysia, Johor Bahru, Malaysia

**Keywords:** cross-cultural adaptation, emotional expressivity, Berkeley expressivity questionnaire, psychometric properties, Chinese students

## Abstract

This study investigates the psychometric properties of the Berkeley Expressivity Questionnaire (BEQ) within a cross-cultural framework, specifically among Chinese international students in Malaysia. Exploratory factor analysis identified a four-dimensional structure that diverges from the original three-factor model. This new structure was subsequently validated through confirmatory factor analysis (*N* = 300), yielding superior model fit indices compared to alternative models, all exceeding conventional benchmarks. The findings highlight significant cross-cultural distinctions, notably in emotional concealment, reflecting the complex interplay between traditional Chinese cultural values and the demands of international educational environments. Detailed analyses indicated that specific adaptations in item wording and context were essential to achieve cross-cultural measurement validity. This research contributes to the methodological discourse on cross-cultural measurement and enriches understanding of emotional expression dynamics among international students. It underscores the importance of culturally responsive adaptations when employing Western-developed assessment tools across diverse populations. The proposed four-dimensional framework offers a refined perspective on emotional expressivity in multicultural educational contexts, providing valuable insights for enhancing the support structures aimed at improving adaptation and psychological well-being for Chinese international students.

## 1 Introduction

### 1.1 Background of the research

Emotional expressivity constitutes a central facet of personality that significantly influences individuals’ capacity for social adjustment and psychological well-being (Lü and Wang, 2012). Research on emotional expression has evolved considerably, beginning with Ekman’s (1993) foundational Basic Emotion Theory, which established that certain emotions represent universal biological phenomena recognizable across cultures through distinct facial expressions such as those conveying anger, happiness, and fear (Ekman, 1993). This theoretical framework provided the essential foundation for subsequent emotional expression research.

Display rules represent culturally determined conventions that govern emotional expression through specific regulatory mechanisms, as established in groundbreaking research on emotional expression across cultures (Ekman et al., 1969). These mechanisms operate through four primary processes: intensification (amplifying emotional displays, such as exaggerated smiling), minimization (reducing negative emotional expressions despite experiencing negative emotions), neutralization (suppressing all emotional indicators, commonly known as maintaining a “poker face”), and substitution (displaying emotions incongruent with one’s actual feelings, such as simulated smiling) (Matsumoto and Kupperbusch, 2001). This framework demonstrates that facial expressions function not merely as spontaneous physiological reactions, but as culturally calibrated responses adjusted to meet contextual requirements and social expectations. Display rules primarily serve to promote interpersonal civility and maintain social cohesion within culturally specific interaction contexts, thereby functioning as essential mechanisms for cultural adaptation and social regulation (Matsumoto et al., 2008). Hence, emotional expressivity, as a core mechanism of human communication and adaptation, has profound implications for individuals’ social functioning, interpersonal relationships, and psychological well-being, making its study critically significant for understanding human behavior and developing effective mental health interventions.

The unique cross-cultural context of Chinese international students pursuing their education in Malaysia presents a compelling case for specialized research attention. While both cultures share important collectivist values, significant cultural disparities in social customs and communication norms between China and Malaysia create distinctive challenges for Chinese international students navigating this educational environment (Shafaei and Razak, 2016). Malaysia represents a diverse multicultural society where Islamic values significantly influence the Malay Muslim majority, emphasizing social cohesion and collective well-being (Haque, 2003). While Malaysia encompasses three major ethnic groups—Malays, Chinese, and Indians—each maintains distinct cultural traditions within a shared national framework, creating a mosaic of parallel value systems rather than a single convergent culture (Selvarajah and Meyer, 2008).

Chinese culture, shaped by Buddhist and Confucian traditions, emphasizes collectivism, family structures, and societal harmony, though these manifest differently from the collectivist elements found in Malay Muslim traditions (Winfield et al., 2000). This cultural plurality creates a unique environment where international Chinese students encounter multiple expressions of collectivism that differ from both their home culture and from each other. Despite these shared collectivist foundations, the specific cultural practices, communication styles, and emotional display rules create a complex environment for emotional expression and regulation that requires sensitive and validated assessment approaches.

When a scale or methodology is designed for a specific population, it is essential to conduct deliberate validation before applying it to different cultural contexts (Beaton et al., 2000). Failure to do so risks inaccuracies in capturing emotional expressivity within the new population (Sousa and Rojjanasrirat, 2011). Despite widespread international adaptation of emotional expressivity measures across diverse cultural contexts, a significant limitation persists regarding validated instruments specifically calibrated for Chinese international students studying in Malaysia navigating cross-cultural environments. While various emotional expressivity measures have been adapted for different cultural contexts, including some Asian cultures, there remains a lack of validated instruments that address the unique psychological challenges faced by Chinese international students in Malaysian universities.

Therefore, validating an instrument like the BEQ for Chinese international students studying in Malaysia is critical to accurately reflect their unique cultural and emotional experiences, as these students navigate a multicultural environment while maintaining their distinct Chinese national identity.

Therefore, the present study aims to adapt the Berkeley Expressivity Questionnaire (BEQ) for Chinese populations and comprehensively evaluate its psychometric properties. Building on these cultural considerations and methodological requirements, this study addresses a critical gap in the literature. This research directly addresses this need by adapting the Berkeley Expressivity Questionnaire for Chinese international students in Malaysian universities—a population experiencing the intersection of Chinese collectivist values and Malaysia’s Islamic-influenced social framework (Ahmad et al., 2024). The nuanced cultural differences between these traditions present distinct challenges for emotional adaptation that significantly impact both academic performance and social integration (Alazzi and Chiodo, 2008).

By validating a culturally appropriate assessment tool for this specific population, this research enhances measurement accuracy and enables more effective interventions to support cross-cultural emotional adjustment. The findings contribute both to theoretical understanding of cultural influences on emotional expression and to practical applications for improving psychological well-being in international educational settings. This study addresses the underexplored cultural context of Chinese international students in Malaysian universities by adapting the Berkeley Expressivity Questionnaire (BEQ) to this population and evaluating its psychometric properties, offering new insights into the emotional expressivity of this group.

## 2 Literature review

### 2.1 Conceptual development of emotional expressivity

The understanding of emotional expressivity has expanded significantly over time, developing from basic theoretical foundations to complex multidimensional constructs. Emotional expressivity was conceptualized as the capacity to communicate emotional experiences through both verbal and nonverbal channels (Kring et al., 1994). Building upon these contributions, the concept was further developed by emphasizing observable behavioral manifestations, including facial expressions, body postures, and specific actions such as smiling, frowning, or abruptly leaving a room, thereby highlighting the behavioral dimensions of emotional expressivity and establishing a more comprehensive framework for understanding this complex construct (Gross and John, 1997). The understanding of emotional expression has advanced significantly through research questioning its fundamental nature and functions. Expressive behaviors were critically examined and proposed to extend beyond mere emotional displays to communicate intentions, anticipated actions, and interpersonal dynamics such as dominance relationships (Fridlund, 1991). This perspective repositioned emotional expressions as sophisticated social signals rather than simple reflections of internal states.

Complementing this conceptual expansion, emotional communication has been empirically demonstrated to transcend visual channels, with research showing that listeners can accurately identify five distinct emotions—anger, fear, happiness, sadness, and tenderness—through vocal cues alone, with recognition accuracy approaching 70% (Hawk et al., 2009). This finding established the importance of vocal expressions in emotional communication and highlighted the multimodal nature of emotional signaling. Collectively, these studies reveal emotional expression as a multifaceted phenomenon shaped not only by biological universals but also by cultural, social, and situational influences (Keltner et al., 2019). Together, they construct a comprehensive framework for understanding the diverse mechanisms and contextual factors that determine how emotions are expressed across various domains and communication channels.

### 2.2 Review of existing scales measuring emotional expressivity

The Affective Communication Test (ACT; Friedman and Riggio, 1981) is a 13-item self-report scale designed to measure nonverbal expressiveness through subscales examining performance skills and interpersonal emotional communication. The instrument employs a 9-point response format with anchors ranging from “Not true of me” (−4) to “Very true of me” (+4). While offering an innovative approach to assessing expressive capabilities, the ACT items often address expressiveness in social gatherings, and general emotional impact on others (Friedman and Riggio, 1981). These may not directly translate to or fully capture the emotional expressivity requirements and challenges that students face in academic environments. The instrument’s design suggests potential limitations in comprehensively addressing the context-specific emotional experiences of students, highlighting the potential need for more targeted measurement approaches tailored to educational environments.

The Positive and Negative Affect Schedule (PANAS) is a widely used instrument that assesses two core dimensions of emotional experience: *positive affect* and *negative affect* (Watson et al., 1988). It measures the specific emotions individuals experience and the extent to which they experience them within a specified time frame (e.g., “in the past week”). Respondents rate each of the 20 items—10 for positive affect and 10 for negative affect—using a 5-point Likert scale ranging from 1 (*Very slightly or not at all*) to 5 (*Extremely*). Importantly, the PANAS captures the *intensity* rather than the *frequency* of emotional experiences, offering a nuanced measure of affective states. The PANAS demonstrates strong psychometric properties, including high internal consistency and low inter-scale correlation. It has also shown good temporal stability over a two-month period, supporting its reliability and validity as a measure of affect (Crawford and Henry, 2004). However, it is important to note that the PANAS primarily assesses *emotional states*—the internal experience of affect—rather than *emotional expressivity*.

Research also indicates that people may experience positive and negative emotions at different intensity levels (Weinfurt et al., 1994). Affect Intensity Measure (AIM) is a 20-item tool that measures how deeply emotions are felt subjectively, utilizing a 6-point Likert scale ranging from 1 (Never) to 6 (Always) (Larsen, 1985). This design quantifies individual differences in emotional intensity across various situations, distinguishing between those who typically experience emotions strongly versus moderately. It features two subscales: positive (11 items, *α* = 0.83) and negative (7 items, α = 0.72), focusing on the internal strength of emotional responses. Importantly, the AIM’s primary limitation is that it does not include measurement of outward emotional expression, specifically assessing how strongly people feel emotions internally.

The Toronto Alexithymia Scale (TAS-20) is a 20-item self-report questionnaire designed to assess alexithymia, a personality construct characterized by difficulties in identifying and describing emotions, as well as externally oriented thinking (Bagby et al., 1994). Respondents rate each item on a 5-point Likert scale, ranging from 1 (strongly disagree) to 5 (strongly agree). While the TAS-20 has demonstrated solid reliability and has been widely validated in clinical and non-clinical populations (Bagby et al., 1994; Bagby et al., 2020), as it was originally developed primarily with adult and clinical samples, the scale may potentially conflate temporary emotional difficulties common among university students with stable alexithymia traits.

The Emotional Expressivity Scale (EES) emerged as a pioneering instrument dedicated to measuring emotional expressivity (Kring et al., 1994). Conceptualized as a unidimensional 17-item self-report tool, the EES evaluates an individual’s general propensity to externalize emotions, transcending specific emotional valence or expression channels. Demonstrating robust psychometric properties, the scale exhibits exceptional internal consistency with alpha coefficients ranging from 0.88 to 0.91 across diverse samples, and a remarkable test–retest reliability of 0.90 over a 4-week period (Kring et al., 1994). The scale’s validity is reinforced by its strong convergence with peer-based assessments of general expressivity and its demonstrated ability to predict emotion-expressive behaviors in controlled laboratory environments.

The Berkeley Expressivity Questionnaire (BEQ) represents a well-established 16-item self-report instrument specifically designed to assess individual differences in emotional expressivity through three empirically validated dimensions: negative expressiveness, positive expressiveness, and impulse strength (Gross and John, 1997; Gross and John, 1995). The instrument employs a 7-point Likert scale (1 = strongly disagree to 7 = strongly agree) to evaluate respondents’ capacity to express emotions. Its comprehensive approach captures both internal emotional experiences and their external manifestations, providing valuable insights into emotional tendencies and behaviors (Dobbs et al., 2007). In terms of reliability, the BEQ exhibits sound internal consistency, with Cronbach’s alpha coefficients ranging from 0.71 to 0.76 for its three subscales. Temporal stability is evidenced by test–retest reliability coefficients ranging from 0.71 to 0.82 over a two-month interval, meeting accepted thresholds for psychological assessment instruments (Gross and John, 1995).

Regarding its factorial structure, confirmatory analyses identified three distinct factors accounting for 33% of the total variance. Factor loadings demonstrated robust item-scale relationships: 0.44 to 0.80 for positive expressivity, 0.37 to 0.68 for negative expressivity, and 0.53 to 0.69 for impulse strength. The subscales show significant positive intercorrelations, with impulse strength correlating at 0.52 with negative expressivity and 0.50 with positive expressivity, while negative and positive expressivity correlate at 0.51 (Gross and John, 1995). The instrument’s validity is supported through multiple lines of evidence. The Berkeley Expressivity Questionnaire demonstrated convergent validity through significant associations with peer ratings of expressivity and its capacity to predict both positive and negative emotional expressions in laboratory settings (Gross and John, 1997). Further validation studies have confirmed its correlation with other established measures of emotional expression (Kring et al., 1994; Gross and John, 1997).

Unlike unidimensional scales such as the EES, the BEQ provides a more nuanced assessment of emotional expression patterns by distinguishing between positive and negative emotional expressivity while simultaneously measuring impulse strength. In addition, the BEQ uniquely incorporates impulse strength as a dimension, which measures the intensity of emotional response tendencies—a crucial component of emotional expression not captured by other measures. The BEQ also demonstrates strong construct validity, evidenced by its high correlation with the Emotional Expressivity Scale (*r* = 0.88) (Gross and John, 1997), indicating that it effectively captures the core construct of emotional expressivity while offering additional dimensional information. Furthermore, the BEQ’s development was firmly grounded in comprehensive theoretical frameworks that integrate both physiological and behavioral components of emotional expression.

### 2.3 Cross-cultural adaptations of the BEQ

The Berkeley Expressivity Questionnaire has demonstrated its cross-cultural applicability through successful adaptations in various cultural contexts. These adaptations typically maintain the three-factor structure of the original instrument while demonstrating good psychometric properties within the target populations. In Turkey (Akın, 2011), the adaptation study confirmed the three-factor structure with adequate internal consistency (Cronbach’s alpha ranging from 0.70 to 0.82) and significant correlations with measures of psychological well-being. The German adaptation similarly retained the original factor structure with acceptable to good internal consistency (Cronbach’s alpha ranging from 0.72 to 0.85) and demonstrated meaningful associations with personality measures (Mohiyeddini et al., 2008). The Japanese adaptation provided evidence for the cross-cultural validity of the BEQ in an East Asian context, though some cultural differences in factor loadings were noted, particularly for items related to negative expressivity (Lin et al., 2016). This finding highlights the importance of cultural sensitivity in emotional expressivity assessment. The Dutch adaptation confirmed the three-factor structure and demonstrated good internal consistency (Cronbach’s alpha ranging from 0.74 to 0.81) and test–retest reliability (Kupper et al., 2020). The Bangladeshi adaptation represents another application in a South Asian context, demonstrating adequate internal consistency (Cronbach’s alpha ranging from 0.68 to 0.77) and meaningful correlations with measures of psychological adjustment among university students (Aktar and Uddin, 2024).

To sum up, the BEQ has demonstrated robust psychometric properties across diverse cultural contexts, having been successfully adapted and validated in various countries. These methodological strengths have earned the BEQ international recognition as a valuable assessment tool for emotional expressivity. The cultural adaptation process typically involves translation, pilot testing, administration to the target population, and thorough evaluation of validity and reliability to ensure the instrument’s effectiveness in new cultural settings (Sousa and Rojjanasrirat, 2011).

These diverse cross-cultural adaptations highlight the BEQ’s versatility and relevance across different cultural contexts while also emphasizing the importance of cultural sensitivity in emotional expressivity assessment. The successful adaptation across multiple cultures provides a strong foundation for the current study’s focus on adapting the BEQ for Chinese international students in Malaysian universities.

## 3 Method

### 3.1 Item development and validation

The Likert scale constitutes one of the most fundamental and widely implemented psychometric instruments across various research domains, including sociology, psychology, information systems, political science, economics, and numerous other fields. Based on comprehensive analysis, the seven-point rating scale is recommended as the most appropriate measurement format for optimal psychometric properties (Taherdoost, 2019). Accordingly, this adapted version maintains the 7-point scale structure consistent with the original Berkeley Expressivity Questionnaire (Gross and John, 1997), ensuring methodological continuity with the established instrument.

Content validity evaluates the extent to which questionnaire items accurately represent their target constructs (Polit and Beck, 2006). Subject matter experts typically conduct this assessment by determining whether an instrument’s items comprehensively cover the measured concept (Bannigan and Watson, 2009). For quantitative assessment, researchers employ the Content Validation Index (CVI). This method requires experts to rate each item on a 4-point relevance scale (1 = not relevant to 4 = highly relevant). Items scored 3–4 are designated “content valid,” while those rated 1–2 are considered “content invalid” (Polit and Beck, 2006). The CVI is calculated by dividing the number of valid items by the total item count. For example, an instrument with 8 of 10 items rated valid would have a CVI of 0.8. Establishing adequate content validity requires meeting a minimum CVI threshold of 0.8, with underperforming items targeted for revision or removal (Yusoff et al., 2019). Content Validity Index of 1.0 indicates that both experts considered all items in the adapted BEQ to be content valid (see Table 1), suggesting strong agreement that the questionnaire items appropriately represent the constructs being measured.

**Table 1:** CVI for adapted BEQ.

Measurement scale	Total number	Expert 1			Expert 2			Overall CVI
		Valid items	Invalid items	CVI	Valid items	Invalid items	CVI	1
Adapted BEQ	16	16	0	1	16	0	1	1

Two educational psychology specialists performed comprehensive reviews that confirmed the questionnaire items accurately reflect the intended constructs. Following these expert evaluations, minor refinements were made to the instrument. The wording of specific items was adjusted and enhanced based on the specialists’ recommendations to improve clarity and conceptual alignment. The items were then translated into Chinese, with linguistic experts conducting additional validation to detect and correct any phrasing inconsistencies. This thorough validation process ensures the instrument demonstrates both measurement precision and cultural appropriateness in assessing support dynamics (Sousa and Rojjanasrirat, 2011).

### 3.2 Pilot study

The pilot study employed Item Response Theory (IRT), specifically the Rasch model, to evaluate the psychometric properties of the instrument (Wright, 1996). This approach converts ordinal data into interval-scale measurements expressed in logits, allowing for more precise assessment of item functioning and producing comparable measurements with defined units (Sondergeld and Johnson, 2014; Boone et al., 2011). Additionally, the Rasch model enables the removal of irrelevant items, identifies items with high accuracy, and detects potential item bias (Cooke and Michie, 1997). ConQuest version 2 software was utilized for the Rasch analysis due to its powerful capabilities in handling a wide range of measurement models and its ability to process both dichotomous and polytomous item responses efficiently. The software provides comprehensive item fit statistics and allows for clear interpretation of results through well-structured output formats. ConQuest is particularly effective for educational and psychological measurement applications, making it suitable for analyzing instruments such as the one examined in this study (Koyuncu and Şata, 2023).

Data from 110 Chinese postgraduate students at Universiti Teknologi Malaysia were analyzed using ConQuest version 2. This sample size aligns with established guidelines for polytomous Rasch models, which recommend approximately 100–200 participants to achieve stable item parameter estimates due to the increased complexity of item scoring with multiple response categories (Briggs and Wilson, 2003). The sample size of 110 participants was determined to be sufficient for obtaining meaningful results in this pilot phase while maintaining adequate statistical power.

To evaluate item fit, Mean Square (MNSQ) values were examined, with acceptable ranges between 0.6 and 1.4 (Bond and Fox, 2007), and T statistics, with values between ±2.0 indicating acceptable fit (Linacre, 1999). These criteria help identify items that may not be functioning as expected within the measurement model. All items demonstrated satisfactory fit to the Rasch model, with weighted and unweighted MNSQ values falling within the acceptable range of 0.6 to 1.4. As shown in Table 2, the weighted MNSQ values ranged from 0.80 to 1.23, while T statistics remained within the recommended threshold of ±2.0 for all items. These results indicate that all items functioned appropriately within the measurement model, contributing meaningful information to the assessment of the targeted construct.

**Table 2:** Item parameter estimates for adapted BEQ.

Item	BEQ		UNWEIGHTED FIT			WEIGHTED FIT		
	Estimate	Error	MNSQ	CI	T	MNSQ	CI	T
1	0.068	0.066	0.99	(0.73, 1.27)	−0.0	1.08	(0.72, 1.28)	0.5
2	−0.037	0.050	1.06	(0.73, 1.27)	0.5	1.09	(0.74, 1.26)	0.7
3	0.040	0.053	1.27	(0.73, 1.27)	1.9	1.23	(0.77, 1.23)	1.8
4	−0.110	0.066	1.04	(0.74, 1.26)	0.4	1.02	(0.72, 1.28)	0.2
5	0.024	0.049	0.99	(0.74, 1.26)	−0.0	0.91	(0.76, 1.24)	−0.7
6	−0.268	0.067	0.79	(0.74, 1.26)	−1.7	0.82	(0.73, 1.27)	−1.4
7	−0.300	0.052	1.12	(0.74, 1.26)	0.9	1.13	(0.74, 1.26)	0.9
8	−0.072	0.048	1.12	(0.74, 1.26)	0.9	1.12	(0.76, 1.24)	1.0
9	0.181	0.049	1.12	(0.74, 1.26)	0.9	1.08	(0.76, 1.24)	0.7
10	0.310*	0.115	1.10	(0.74, 1.26)	0.8	1.09	(0.73, 1.27)	0.7
11	−0.369	0.052	0.94	(0.74, 1.26)	−0.4	0.97	(0.75, 1.25)	−0.2
12	−0.171	0.052	0.79	(0.74, 1.26)	−1.6	0.80	(0.75, 1.25)	−1.7
13	−0.131	0.050	0.82	(0.74, 1.26)	−1.4	0.83	(0.75, 1.25)	−1.4
14	0.524	0.050	1.10	(0.74, 1.26)	0.8	1.08	(0.76, 1.24)	0.7
15	0.352*	0.115	1.07	(0.74, 1.26)	0.6	1.01	(0.75, 1.25)	0.1
16	−0.042*	0.111	0.91	(0.74, 1.26)	−0.7	0.92	(0.76, 1.24)	−0.6

### 3.3 Participants and procedure

The study recruited 300 Chinese international postgraduate students from a Malaysian public university, surpassing the minimum sample size requirements for robust statistical analysis. Following the recommended 1:10 item-to-sample ratio for structural equation modeling (SEM) (Kline, 2017), with 16 measurement items in the Berkeley Expressivity Questionnaire, the minimum required sample was 160 participants. Initially, 330 questionnaires were collected, yielding a final analytical sample of 300 participants. Employing Mahalanobis distance (D^2^) with a *p* < 0.001 chi-square distribution threshold (Mahalanobis, 2018; Hair, 2011). We systematically identified and eliminated 10 multivariate outliers. Complementary techniques of standard deviation analysis and boxplot visualization facilitated the exclusion of 3 additional anomalous observations. A rigorous screening process further detected straight-lining patterns across survey items (Reuning and Plutzer, 2020), leading to the removal of 17 methodologically compromised samples, totaling 30 samples removed from the initial dataset.

A random data-splitting procedure was implemented to create independent subsamples for exploratory and confirmatory factor analyses. A ratio of 5–10 participants per item is generally recommended for exploratory factor analysis (EFA) to achieve stable factor solutions (Hair, 2011). Accordingly, the total sample (*N* = 300) was randomly partitioned into an EFA subsample (*n* = 100) and a CFA subsample (*n* = 200). This partitioning was executed through a random number generation process in SPSS, followed by case sorting based on the random values, and subsequent assignment of cases to respective analytical groups. This methodological approach eliminates the potential for capitalization on chance that might occur when conducting both EFA and CFA on the same dataset, thereby enhancing the validity of the factor structure identification and subsequent confirmation processes (Hair, 2011).

The data collection process followed formal institutional protocols, beginning with official authorization via a formal letter from the Faculty of Educational Sciences and Technology. The research procedures adhered to structured methodological guidelines (Sng et al., 2016) and received requisite permissions from relevant institutional review authorities to address potential ethical implications. The investigation rigorously maintained the principle of autonomous participation. This included obtaining informed consent without coercion and ensuring participants possessed comprehensive awareness regarding their research involvement (Felzmann, 2009; Ederio et al., 2023). The researchers maintained strict adherence to the ethical imperative of safeguarding participant rights while preserving the scientific and reputational integrity of their affiliated academic institution (Nneoma et al., 2023).

### 3.4 Data analysis procedure

Data analysis was conducted using several specialized statistical software packages. The exploratory factor analysis (EFA) was performed using IBM SPSS Statistics (Version 29.1.0), enabling the initial identification of the factor structure underlying the Berkeley Expressivity Questionnaire responses among Chinese international students in Malaysian universities. SPSS was selected for this phase due to its robust capabilities in handling multivariate analyses and its widespread acceptance in psychological research (Pituch and Stevens, 2015).

Mplus 8 (Version 1.6.1) was employed to conduct confirmatory factor analysis (CFA) for testing three distinct measurement models: the original three-factor model (M1), the EFA-derived three factor model (M2), and a revised four-factor model (M3). Mplus was specifically chosen for its superior handling of latent variable modeling and its flexibility in testing competing structural models (Byrne, 2013). To establish the reliability and validity of the four-dimensional construct, including assessment of convergent and discriminant validity, SmartPLS 4 (Version 4.1.0.9) was utilized to evaluate the inner model fit. SmartPLS was selected for its particular strengths in evaluating measurement models and its capacity to handle complex path relationships without strict distributional assumptions (Hair, 2011). This comprehensive analytical approach allowed for rigorous testing of the psychometric properties of the adapted BEQ in this cross-cultural context (Byrne and Van de Vijver, 2010). This comprehensive analytical approach allowed for rigorous testing of the psychometric properties of the adapted BEQ in this cross-cultural context.

## 4 Results

### 4.1 Demographic information

The demographic analysis of the 300-participant study reveals a near-equal gender distribution, with females constituting 55.0% (165 individuals) and males 45.0% (135 individuals). The age stratification highlights a concentration of middle-aged (26–40) participants representing 54.7% (164 individuals), accompanied by 24.3% (73) young adults (18–25) and 21.0% (63) adults (41 and above). Educational background reveals a significant stratification, with a substantial majority of 65.7% (197 participants) attaining advanced doctoral-level qualifications, while the remaining 34.3% (103) hold master’s degrees, indicating a research sample with robust academic credentials. This demographic composition—characterized by its middle-aged, predominantly female, and relatively well-educated profile—provides a comprehensive foundation for the research investigation.

### 4.2 Exploratory factor analysis

Although exploratory factor analysis (EFA) and confirmatory factor analysis (CFA) both examine underlying factor structures in data, they fulfill different research functions: EFA focuses on building theories, while CFA concentrates on testing theoretical propositions (Matsunaga, 2010). Prior to implementing factor analysis, it is essential to verify the suitability of the research data for this analytical approach (Hair, 2011). Since the Berkeley Expressivity Questionnaire (BEQ) is a well-established measure developed in Western cultural contexts and validated across various populations (Mohiyeddini et al., 2008; Kupper et al., 2020), we first employed CFA to examine the applicability of its original three-factor structure among Chinese international students from mainland China. This approach aligns with standard methodological procedures in cross-cultural adaptation research, which prioritizes evaluating the original instrument structure in new cultural contexts (Sousa and Rojjanasrirat, 2011).

The CFA results indicated poor model fit for the original model (M1): χ^2^/df = 7.778, *p* < 0.0001, CFI = 0.789, TLI = 0.740, SRMR = 0.166, RMSEA = 0.184 (90% CI: 0.170–0.198) (see Table 3). These fit indices fell substantially below commonly accepted thresholds for adequate model fit (CFI and TLI > 0.90, SRMR < 0.08, RMSEA < 0.08) (Bentler, 1990; Hu and Bentler, 1999), suggesting that the original factor structure of the BEQ may not be directly applicable to Chinese international students in the Malaysian context. Thus, the poor fit from the CFA provided clear methodological justification for subsequently conducting exploratory factor analysis (EFA) (Hurley et al., 1997). Given that the original factor structure proved inadequate, we needed to explore the potential latent factor structure of emotional expressivity in this specific cultural context through EFA.

**Table 3:** Evaluation of the outer measurement model.

Model	χ2/df	*p*-value	CFI	TLI	SRMR	RMSEA		
						estimate	90 percent C.I.	Probability RMSEA <= 0.05
M1	7.778	0.0000	0.789	0.740	0.166	0.184	0.170–0.198	0.000
M2	6.273	0.0000	0.836	0.798	0.097	0.162	0.148–0.177	0.000
M3	1.176	0.1476	0.995	0.993	0.029	0.030	0.000–0.053	0.922

Principal Axis Factoring (PAF) was employed as the extraction method in this analysis. Unlike Principal Component Analysis, PAF focuses specifically on common variance instead of total variance, making it especially suitable for identifying underlying latent constructs (Becker et al., 2013; Fabrigar et al., 1999). This approach requires fewer assumptions about data distribution, providing better capability to handle psychological measurement data that may not conform to multivariate normality assumptions (Flora and Curran, 2004).

The analysis was based on the correlation matrix, and factors were extracted using Kaiser’s criterion (eigenvalues > 1). The Varimax method, an orthogonal rotation technique, was employed for rotation purposes. This methodological approach systematically optimizes the variance distribution across factor loadings, thereby generating a solution in which individual factors demonstrate robust correlations with specific, discrete variable subsets (Kline, 2014). The resultant factor structure exhibits both parsimony and interpretive clarity. It is noteworthy that varimax rotation functions under the theoretical premise that factors maintain independence from one another, preserving orthogonality within the factorial solution (Yong and Pearce, 2013). Such a presentation framework significantly augments researchers’ capacity to identify item-factor relationships and interpret the emergent factorial architecture with enhanced analytical precision (Osborne, 2015).

Initial analysis validated the Berkeley Expressivity Questionnaire (BEQ) data’s suitability for factor analysis through two key statistical indicators. The Kaiser-Meyer-Olkin sampling adequacy index yielded a coefficient of 0.883 (see Table 4), falling within the “meritorious” range (0.80–0.90) and exceeding the minimum threshold of 0.6 (Nkansah, 2018). Simultaneously, Bartlett’s Test of Sphericity produced a statistically significant result (χ^2^ = 1379.813, df = 120, *p* < 0.001), rejecting the null hypothesis that the correlation matrix approximates an identity matrix. These complementary metrics confirmed adequate sampling properties and sufficient variable intercorrelations, substantiating the appropriateness of factor analytic techniques for identifying the latent structure underlying the BEQ (Kaiser, 1974; Costello and Osborne, 2005).

**Table 4:** KMO and Bartlett’s test of adapted BEQ.

Kaiser-Meyer-Olkin measure of sampling adequacy.		0.883
Bartlett’s Test of Sphericity	Approx. Chi-Square	1379.813
	df	120
	Sig.	0.000

**Figure 1:**
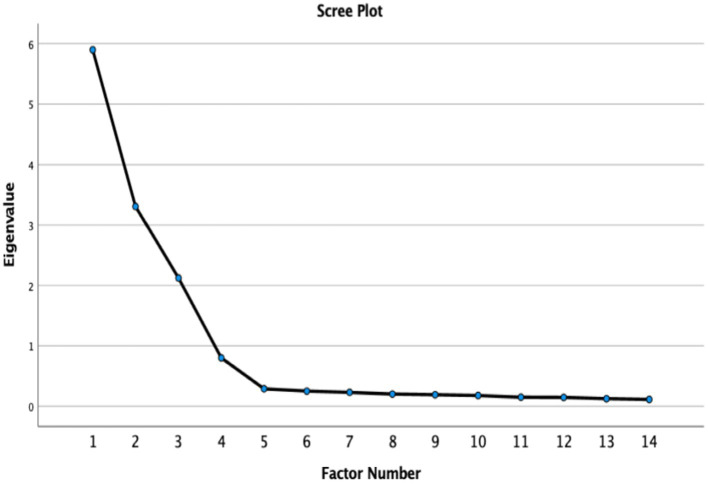
Scree Plot of adapted BEQ.

The scree plot provides clear evidence supporting an alternative solution for the Berkeley Expressivity Questionnaire (see Figure 1). The first three factors show substantial eigenvalues, exceeding Kaiser’s criterion of 1.0 (Kaiser, 1960). However, a distinct “elbow” or “gap” appears after the fourth factor, with subsequent eigenvalues falling below 0.5 and the curve flattening considerably (Zhu and Ghodsi, 2006). This pattern partially satisfies the Kaiser criterion (supporting a three-factor solution) while Cattell’s scree test (Cattell, 1966) might suggest a four-factor structure due to the visible elbow. The slight divergence between these results and the superior fit indices obtained through confirmatory factor analysis provides evidence that careful consideration is needed when determining whether a three-factor or four-factor model is the most appropriate representation of emotional expressivity within this specific cultural context.

### 4.3 Item removal and immigration

As previous mentioned, the Berkeley Expressivity Questionnaire (BEQ) was originally conceptualized with a tripartite structure encompassing Positive Expressivity (PE), Negative Expressivity (NE), and Impulse Strength (IS), with the latter dimension assessing generalized emotional intensity irrespective of valence (Gross and John, 1995). Similarly, psychometric analysis conducted in the present study initially yielded a three-factor framework (refer to Table 5).

**Table 5:** Rotated factor matrix^a^.

Items	Factor		
	1	2	3
NI1(is1)	−0.071	**0.885**	−0.014
NI2 (is4)	−0.053	**0.907**	−0.01
NI3(is5)	−0.045	**0.893**	−0.032
NI4(is6)	−0.11	**0.890**	−0.063
NE1(ne2)	0.157	−0.041	0.861
NE2 (ne3)	0.111	−0.051	**0.839**
NE3(ne4)	0.15	0.05	**0.879**
NE4 (ne5)	0.234	−0.035	**0.836**
PE1(pe1)	0.628	−0.437	0.331
PE2(pe3)	0.647	−0.409	0.304
PE3(pe4)	0.653	−0.332	0.395
PI1(is2)	**0.844**	0.05	0.161
PI2(is3)	**0.864**	0.001	0.056
PI3(pe2)	**0.870**	0.011	0.14

The bold values in the rotated factor matrix represent the highest factor loadings for each item across the three factors. This formatting convention highlights which factor each item loads most strongly on, making it easier to identify the underlying structure of the Berkeley Expressivity Questionnaire.

The EFA results suggested both three-factor and four-factor structures as viable alternatives (refer to Table 6). Factor 1 integrates two conceptually distinct types of items: item *is2 is3* and item *pe2* exhibiting robust factor loadings (0.844–0.870) and items *pe1 pe 3* and item *pe4* demonstrating comparatively moderate loadings (0.628–0.653). Notably, PE items (items: *pe1*, *pe3* and *pe4*) display substantial cross-loadings across multiple factors, specifically negative correlations with Factor 2 (−0.332 to −0.437) and positive correlations with Factor 3 (0.304 to 0.395). These loading patterns suggest a meaningful psychometric distinction between these two item clusters within Factor 1, indicating the potential value of treating them as separate constructs.

**Table 6:** Items recoding.

Subscale item	
Factor 1 positive expression	
PE1	*pe1*: Whenever I feel positive emotions, people can easily see exactly what I am feeling while studying abroad. 在海外留学期间,每当我感到愉快时, 大家能感知到我的情绪。
PE2	*pe3*: When I’m happy, my feelings show while studying abroad. 在海外留学期间, 每当我感到愉快时, 我的情绪会表现出来。
PE3	*pe4*: I am an emotionally expressive person while studying abroad. 在海外留学期间, 我是一个情感表达丰富的人。
Factor 2 negative expression	
DELETED	*ne1*: People often do not know what I am feeling while studying abroad. 在海外留学期间,人们常常不知道我的想法。
NE1	*ne2*: It is difficult for me to hide my fear while studying abroad. 在海外留学期间,我很难隐藏害怕的情绪。
NE2	*ne3*: I’ve learned it is better to suppress my anger than to show it while studying abroad. 在海外留学期间, 我认为压抑愤怒比表现出来更好。
NE3	*ne4*: No matter how nervous or upset I am, I tend to keep a calm exterior when studying abroad. 无论我有多不安或沮丧, 我都会尝试保持冷静的外表。
NE4	*ne5:* Whenever I feel negative emotions, people around me can easily see exactly what I am feeling while studying abroad. 在海外留学期间, 每当我有消极情绪时, 周围的人很容易看出我的感受。
DELETED	*ne6:* What I’m feeling is written all over my face while studying abroad. 在海外留学期间, 我的情感都写在脸上, 一目了然。
Factor 3 positive intensity	
PI1	*is2*: My body reacts very strongly to emotional situations while studying abroad. 在海外留学期间,我的身体对情绪化的情境反应非常强烈
PI2	*is3*: I have strong emotions while studying abroad. 在海外留学期间, 我的情感非常强烈。
PI3	*pe2*: I laugh out loud when someone tells me a joke that I think is funny while studying abroad. 在海外留学期间,当别人讲了一个好笑的笑话时, 我会大声笑出来。
Factor 4 negative intensity	
NI1	*is1*: I sometimes cry during sad movies while studying abroad. 在海外留学期间, 在观看悲伤的电影时, 我有时会哭出来。
NI2	*is4*: I am sometimes unable to hide my feelings, even though I would like to while studying abroad. 在海外留学期间, 有时即使我想隐藏自己的情绪, 也无法做到。
NI3	*is5*: There have been times when I have not been able to stop crying even though I tried to stop while studying abroad. 在海外留学期间, 有些时候即使我努力想停止哭泣, 也无法做到。
NI4	*is6*: I experience my emotions very strongly while studying abroad. 在海外留学期间, 我能清楚的感受到自己的情绪。

Theoretical frameworks substantiate this differentiation, as expressivity (pertaining to the frequency and willingness to manifest emotions) and intensity (concerning the magnitude of emotional experience) constitute distinct components of emotional regulation, notwithstanding their contextual interrelationships. Rigorous psychometric analyses demonstrate that a four-factor model more precisely represents the multidimensional complexity of emotional experience while providing superior measurement integrity and construct validity. Section 4.4.1 will elaborate comprehensively on the empirical superiority of the four-factor model (distinguishing between PE and PI) over the three-factor alternative (refer to Table 6). The confirmatory factor analysis and structural equation modeling results reveal that the four-factor model exhibits significantly enhanced fit indices across multiple statistical parameters. Moreover, as detailed in section 4.4.2, the Fornell-Larcker criterion and Heterotrait-Monotrait (HTMT) ratio analyses furnish additional empirical confirmation of discriminant validity between the PE and PI constructs, further validating their conceptual and statistical distinctiveness.

Last but not the least, Items *ne1* and *ne6* demonstrated problematic psychometric properties in our analysis. Item *ne1* showed borderline acceptable loading on its intended factor 1 (0.303) but displayed substantial cross-loading on factor 3 (0.392). Similarly, item *ne6* loaded strongly on both factor 3 (0.483) and its theoretically assigned factor 1 (0.476), indicating considerable construct ambiguity. According to established methodological guidelines, items with significant loadings across multiple factors compromise measurement model clarity, with researchers typically removing variables exhibiting cross-loadings above 0.3 to preserve conceptual distinctiveness (Hair, 2011).

Further content analysis of these items revealed additional concerns. Upon examining the statement content of *ne1*: ‘People often do not know what I am feeling while studying abroad’ (在海外留学期间,人们常常不知道我的想法) and *ne6*: ‘What I’m feeling is written all over my face while studying abroad’ (在海外留学期间, 我的情感都写在脸上, 一目了然) (refer to Table 6), we identified that both express more neutral experiences compared to other NE factor items. These statements appear to capture generalized perceptions rather than specifically addressing the core theoretical dimensions of the construct being measured. Based on both statistical evidence and conceptual analysis within our cross-cultural adaptation framework, we determined that removing these two items would enhance the scale’s measurement precision and construct validity.

To sum up, exploratory factor analysis demonstrated a notable restructuring of the instrument’s dimensional framework. The original Impulse Strength construct bifurcated into two distinct factors—Negative Intensity and Positive Intensity—offering enhanced differentiation between the intensity of positive and negative emotional experiences. And Item *pe2* (“I laugh out loud when someone tells me a joke that I think is funny while studying abroad”) notably migrated from Positive Expressivity to Positive Intensity.

### 4.4 Confirmatory factor analysis

#### 4.4.1 Outer model evaluation

Researchers have emphasized the importance of employing confirmatory factor analysis (CFA) to evaluate whether measurement instruments and their constituent items function according to theoretical expectations (Artino et al., 2014). Specifically, a comprehensive assessment was conducted examining multiple indicators of psychometric adequacy, including (1) model fit indices (χ^2^/df, *P*-Value, CFI, TLI, SRMR, RMSEA) (Sathyanarayana and Mohanasundaram, 2024), (2) convergent validity (average variance extracted) and reliability coefficients (Cronbach’s alpha and composite reliability) for each measurement subscale (Haji-Othman and Yusuff, 2022), (3) discriminant validity through Fornell-Lacker Criterion and HTMT (Fornell and Larcker, 1981).

Table 3 provides a detailed comparison of fit indices across three competing models. According to established research standards, models exhibiting good fit generally display a χ^2^/df ratio less than 3.0, with more stringent criteria recommending thresholds of 2.0 or 1.0 (Byrne, 2013; Hu and Bentler, 1999), which validate the four-factor structure’s superiority (M3). Furthermore, a chi-square test *p*-value greater than 0.05 indicates that differences between the model and empirical data are statistically non-significant (Kline, 2023).

For incremental fit indices, Tucker-Lewis Index (TLI) and Comparative Fit Index (CFI) values should reach a minimum threshold of 0.90, with values of 0.95 or higher preferred (Hu and Bentler, 1999). Regarding residual-based indices, Standardized Root Mean Square Residual (SRMR) values below 0.08 are considered acceptable, with measurements below 0.05 demonstrating excellent fit (Hu and Bentler, 1999). Furthermore, when Probability (RMSEA ≤ 0.05) exceeds 0.50, there exists a greater likelihood of model adequacy, with values approaching 1.00 suggesting near certainty of good fit (MacCallum et al., 1996).

The original three-dimensional model (M1) demonstrates pronounced inadequacy across all psychometric criteria: the normalized chi-square ratio (χ^2^/df = 7.778) substantially exceeds conventional thresholds, incremental fit indices (CFI = 0.789; TLI = 0.740) fall markedly below acceptable parameters, and absolute fit indices (SRMR = 0.166; RMSEA = 0.184, 90% CI [0.170, 0.198]) significantly transgress established limits. The probability of RMSEA ≤ 0.05 being zero further corroborates the model’s empirical inadequacy, indicating that this conceptualization fundamentally fails to capture the underlying construct structure.

Model 2 (M2) shows improved yet still unsatisfactory fit parameters: χ^2^/df = 6.273 remains substantially above the criterion of 3, CFI = 0.836 and TLI = 0.798 fail to reach the acceptable level of 0.90, while SRMR = 0.097 and RMSEA = 0.162 (90% CI [0.148, 0.177]) both exceed their respective thresholds. These findings suggest that while M2 offers incremental improvement over the Model 1, it remains insufficient for adequately capturing the latent dimensional structure.

The third model (M3) exhibits exemplary fit characteristics across all conventional psychometric indices. The normalized chi-square ratio (χ^2^/df = 1.176) falls substantially below critical thresholds, while the non-significant chi-square test (*p* = 0.1476) indicates statistical congruence between model-implied and observed covariance matrices. Incremental fit indices (CFI = 0.995; TLI = 0.993) exceed conservative benchmarks, and absolute fit indices (SRMR = 0.029; RMSEA = 0.030, 90% CI [0.000, 0.053]) demonstrate near-optimal values. The probability of RMSEA ≤ 0.05 (0.922) further corroborates the precision of model specification. This constellation of superior fit statistics provides robust empirical validation for M3’s representation of the underlying data structure.

Based on these established criteria, the comparative analysis of our three models clearly demonstrates the superiority of Model 3 (the four-factor structure) across multiple fit indices. The statistical evidence strongly supports this four-factor conceptualization over both alternative models, confirming its capacity to accurately represent the underlying dimensional structure of the data. These findings offer compelling statistical support for conceptualizing emotional expressivity and intensity as a four-dimensional construct. The results confirm that differentiating between expression and intensity dimensions along positive and negative valence axes provides the most accurate representation of the observed data structure. This four-factor model substantially enhances explanatory power beyond conventional approaches by capturing fundamental distinctions in emotional organization that previous frameworks have overlooked.

#### 4.4.2 Inner model evaluation

The average variance extracted (AVE) values for all latent constructs substantially exceed the recommended threshold of 0.50 (Fornell and Larcker, 1981), satisfying established criteria for convergent validity. Furthermore, both Cronbach’s alpha (*α*) and composite reliability (CR) coefficients for all measured variables considerably surpass the conventional 0.70 benchmark, thereby fulfilling statistical requirements for reliability and internal consistency (Sarstedt et al., 2021). To address potential statistical complications, variance inflation factor (VIF) values were also scrutinized. VIF values below 5 were deemed to represent acceptable multicollinearity levels, while values exceeding 10 indicated significant multicollinearity issues (Sarstedt et al., 2021; Becker et al., 2015).

Rigorous psychometric evaluation of the four-factor emotional expressivity model demonstrates exemplary measurement properties (see Table 7). The Negative Expressivity dimension (NE) exhibits robust reliability coefficients (*α* = 0.918, rho_A = 0.921, rho_C = 0.942) and strong convergent validity (AVE = 0.803), with substantial factor loadings (0.867–0.911) and satisfactory collinearity diagnostics (VIF = 2.51–3.424). Similarly, the Positive Impulse Strength construct (PI) manifests excellent reliability indicators (*α* = 0.928, rho_A = 0.928, rho_C = 0.954) and convergent validity (AVE = 0.874), accompanied by high factor saturations (0.930–0.942) and acceptable multicollinearity indices (VIF = 3.512–4.367). The Negative Impulse Strength factor (NI) yields impressive reliability metrics (α = 0.936, rho_A = 0.941, rho_C = 0.954) and convergent validity (AVE = 0.839), with strong item loadings (0.908–0.924) and acceptable variance inflation factors (VIF = 3.592–4.234). The Positive Expressivity dimension (PE) demonstrates superior reliability coefficients (α = 0.945, rho_A = 0.946, rho_C = 0.965) and convergent validity (AVE = 0.902), with the most robust factor loadings (0.948–0.951) and acceptable, albeit marginally elevated, multicollinearity indicators (VIF = 4.811–5.338).

**Table 7:** Evaluation of the outer measurement model and VIF for multicollinearity.

Abbr.	Outer loading	α	C.R (rho_a)	C.R (rho_c)	AVE	VIF
NE		0.918	0.921	0.942	0.803	
NE1(ne2)	0.899					3.036
NE2(ne3)	0.867					2.51
NE3(ne4)	0.906					3.424
NE4(ne5)	0.911					3.294
PI		0.928	0.928	0.954	0.874	
PI1(is2)	0.930					3.512
PI2 (is3)	0.933					4.034
PI3(pe2)	0.942					4.367
NI		0.936	0.941	0.954	0.839	
NI1(is1)	0.912					3.592
NI2(is4)	0.924					4.097
NI3(is5)	0.920					4.234
NI4(is6)	0.908					3.56
PE		0.945	0.946	0.965	0.902	
PE1(pe1)	0.950					5.338
PE2(pe3)	0.951					5.08
PE3(pe4)	0.948					4.811

Our analysis revealed that one variable exhibited a VIF value of 5.338, which slightly exceeds the more conservative threshold of 5 but remains well below the critical threshold of 10. This marginally elevated VIF value warrants attention but does not necessarily indicate problematic multicollinearity that would compromise the validity of our findings. These comprehensive findings provide compelling evidence for the psychometric integrity of the four-factor structure that emerged during the cross-cultural adaptation of the Berkeley Expressivity Questionnaire.

Researchers in this field have proposed that discriminant validity is only confirmed when a theoretical construct accounts for more variance in its own measurement indicators than it shares with other constructs in the same model (Sarstedt et al., 2021). To satisfy this methodological requirement empirically, two statistical conditions must be fulfilled: (1) for any latent variable, the square root of its average variance extracted (AVE) should be larger than its correlation coefficients with other latent variables in the model (Fornell and Larcker, 1981), and (2) any latent variable’s average variance extracted (AVE) must exceed its maximum shared squared variance (MSV) with other constructs in the model (Hair, 2011; Sarstedt et al., 2021).

As shown in Table 8, discriminant validity assessment through both Fornell-Larcker Criterion and Heterotrait-Monotrait (HTMT) ratio analyses provides robust confirmation of the four-dimensional structure underlying the emotional expressivity framework. The Fornell-Larcker criterion results demonstrate that square roots of Average Variance Extracted for each construct (NE = 0.896, NI = 0.916, PE = 0.950, PI = 0.935) exceed their corresponding inter-construct correlations across all dimensions, fulfilling the primary statistical requirement for discriminant validity.

**Table 8:** Discriminant validity-Fornell-Lacker criterion and HTMT ratio results.

	Fornell-Lacker Criterion				HTMT Results			
	1-NE	2-NI	3-PE	4-PI	1-NE	2-NI	3-PE	4-PI
1-NE	**0.896**							
2-NI	−0.15	**0.916**			0.158			
3-PE	0.245	−0.207	**0.950**		0.236	0.219		
4-PI	0.119	−0.011	0.484	**0.935**	0.128	0.043	0.516	

The bold formatting serves to visually highlight these critical reference values that must be compared against all corresponding off-diagonal correlations to verify discriminant validity.

Examination of inter-factor relationships reveals noteworthy patterns: the moderate positive association between PE and PI (*r* = 0.484) indicates a natural relationship between positive emotional expression and intensity components, while the slight negative correlation between NE and NI (*r* = −0.150) suggests these constructs remain distinct despite their theoretical relatedness. The HTMT analysis yields additional confirmatory evidence, with all ratio values (ranging from 0.043 to 0.516) falling substantially below the conservative threshold of 0.85 (Sarstedt et al., 2021), thereby providing further statistical support that each construct captures unique variance.

These convergent analytical results provide empirical confirmation that the four latent constructs—Negative Expressivity, Negative Intensity, Positive Expressivity, and Positive Intensity—constitute conceptually and statistically distinct dimensions within the emotion processing framework. Discriminant validity assessment demonstrates that each construct’s Average Variance Extracted (AVE) value exceeds its corresponding Maximum Shared Squared Variance (MSV), confirming that each construct shares more variance with its associated indicators than with other constructs in the model (Hair, 2011). This relationship between AVE and MSV values serves as a critical indicator that the measurement model successfully distinguishes between theoretically separate constructs. Based on this comprehensive evaluation, the discriminant validity of all measured variables in this study meets both adequacy standards and statistical requirements, thereby validating the independence of the proposed four-dimensional structure.

## 5 Discussion

Beyond the two items exhibiting ambiguity (items *ne1* and *ne6*), additional evidence emerged supporting emotional intensity’s differentiation into a separate dimension. One potential explanation is that Western conceptualizations may approach emotional intensity as a unified construct (Gross and John, 1997; Gross and John, 1995), whereas the current sample reveals a more refined distinction between positive and negative emotional intensity. Significantly, the evolution from the original three-factor model to four-factor structure highlights cultural particularities in emotional experience and expression. The distinct separation of emotional intensity based on valence may indicate culture-specific approaches to processing positive versus negative emotions.

As items *ne1* (“People often do not know what I am feeling while studying abroad”/“在海外留学期间, 人们常常不知道我的想法”) and *ne6* (“What I’m feeling is written all over my face while studying abroad”/“在海外留学期间, 我的情感都写在脸上, 一目了然”) demonstrated ambiguous factor loadings, significant conceptual divergence emerges in the cross-cultural interpretation of emotional expressivity constructs. Item *ne1* showed relatively low loading on its intended Negative Expressivity factor (0.416) with unexpected cross-loading (0.405) on Positive Expressivity, while item *ne6* exhibited slightly higher loading on Positive Expressivity (0.488) rather than its intended Negative Expressivity dimension (0.460).

Western psychological frameworks categorize emotional inhibition as negative expressivity (Gross and Cassidy, 2019), whereas Chinese cultural norms fundamentally reconceptualize such behaviors. Chinese respondents predominantly interpret emotional restraint as culturally congruent and adaptive behavior rather than as an expressive deficiency (Peng, 2002). In Chinese cultural contexts, the demonstration of emotional privacy signifies appropriate emotional regulation competency rather than expressive inadequacy (Wei et al., 2013). Empirical literature documents that emotional concealment and restraint are valorized as virtuous characteristics, while overt emotional manifestation is frequently characterized as frivolous, developmentally immature, or indicative of unreliability (Peng, 2002; Man et al., 2024).

In traditional Chinese culture, emotional restraint is considered virtuous (Wei et al., 2013; Gross and Cassidy, 2019), allowing ‘concealment of feelings’ to be simultaneously interpreted as demonstrating positive self-regulation and, contextually, as negative emotional inaccessibility. Likewise, while ‘transparent emotional display’ typically represents inadequate control within Western paradigms (Matsumoto et al., 2001), Chinese cultural frameworks may view such expressivity either positively as authentic communication or negatively as developmental immaturity, depending on situational appropriateness (Man et al., 2024). This cultural valuation differential provides explanatory power regarding the ambiguous factor loadings and cross-loading patterns observed in cross-cultural applications of expressivity measurement instruments, particularly when Western-developed assessments are administered to East Asian populations without sufficient cultural adaptation.

A possible interpretation of this migration-related item *pe2* suggests that in Chinese cultural contexts, laughter may serve as a crucial marker of emotional intensity rather than simply casual expression. This finding aligns with European Americans value high-arousal positive emotions (excitement) while Hong Kong Chinese prefer low-arousal positive emotions (calmness) (Tsai et al., 2006). This empirical evidence suggests that in Chinese contexts, uninhibited laughter represents significant emotional intensity rather than casual expression, explaining why laughter-related items cluster with emotional intensity dimensions in our factor analysis.

Disappointment manifests universally across cultures, though significant cultural norms govern its appropriate expression (Mesquita and Frijda, 1992). In Chinese cultural traditions, proper personhood involves interdependence and relationship maintenance, requiring individuals to anticipate others’ expectations and adjust their personal preferences, beliefs, and behaviors accordingly. This cultural emphasis on social harmony and self-improvement forms a core aspect of identity development (Li, 2005; Parmar et al., 2004). Additionally, cultural expectations include respecting established social norms and traditions, accepting hierarchical positioning, engaging in self-criticism when failing to meet collective expectations, and taking corrective action to align one’s conduct with these normative standards (Matsumoto et al., 2010; Cui et al., 2022). These cultural values create a framework where emotional regulation serves important social functions rather than representing expressive deficiencies. Within Chinese cultural contexts, emotional restraint and composure are valued as essential indicators of personal maturity and social competence (Chen and Swartzman, 2001; Russell and Yik, 1996). This cultural framework leads individuals to perceive emotional concealment as an adaptive rather than problematic behavior.

This interpretation based on affect valuation is further contextualized when we consider the broader cultural frameworks that govern social interactions in Chinese society. Another perspective emerges from a cultural framework where communication adheres to hierarchical structures based on seniority and authority (Sun, 2016; Fang and Faure, 2011). Confucian principles, which emphasize maintaining appropriate social decorum, have historically diminished the cultural significance of humor (Dong Yue, 2010), while prioritizing emotional restraint captured in the proverb “Talking is silver, silence is gold” (Au and Yeung, 2014). These differences in emotional valuation extend beyond personal preferences into the foundational cultural norms governing Chinese social interaction. Examining the underlying cultural mechanisms reveals that uninhibited laughter constitutes a breach of established communicative conventions, potentially indicating an individual’s difficulty in navigating the subtle requirements of emotional control and social propriety. Scholarly investigations into the nuanced relationship between Chinese cultural traditions and humor expression have demonstrated that excessive laughter may be interpreted as contradicting Confucian ideals of moderation and self-restraint (Yue, 2011). Such culturally specific interpretations markedly influence psychometric factor structures, illustrating how emotional constructs manifest differently across cultural contexts and necessitate appropriate measurement adaptations to maintain validity.

Likewise, the item “*is4*: I am sometimes unable to hide my feelings, even though I would like to while studying abroad” (在海外留学期间, 有时即使我想隐藏自己的情绪, 也无法做到) showed statistical loading predominantly onto the Negative Intensity (NI) factor, suggesting it was interpreted as specifically addressing negative emotional intensity. This loading pattern likely reflects culturally specific interpretive frameworks prevalent in Chinese society, which emphasizes regulation of negative emotional expression in public domains (Russell and Yik, 1996). This regulatory emphasis may intensify in international environments where individuals often function as cultural representatives. In such contexts, the inability to modulate emotional display appears particularly significant when emotions are negatively valenced, while positive emotional expressions may face less stringent cultural restrictions (Wei et al., 2013). The observed factor structure thus appears to reflect a culturally contingent conceptualization of emotional regulation wherein valence becomes an important determinant of the construct’s meaning and social implications, rather than representing a measurement anomaly.

Similarly, item *is6*: ‘I experience my emotions very strongly while studying abroad’ demonstrated strong loading (0.878) on the Negative Intensity factor, with negligible cross-loadings on other dimensions. Though the wording itself maintains emotional neutrality—not specifically indicating positive or negative emotional valence—this clear classification aligns with established patterns in cross-cultural adaptation research, where heightened emotional awareness often correlates with adaptation challenges in international educational environments (Popadiuk and Arthur, 2004; Stamp et al., 2015).

Research consistently demonstrates that international students facing cultural differences, language barriers, and social disconnection commonly report intensified emotional responses predominantly negative in nature, including anxiety, isolation, and homesickness (Wang and Frank, 2002; Rajapaksa and Dundes, 2002). Specifically for Malaysia, a comprehensive investigation conducted by Universiti Teknologi Malaysia identified 11 distinct challenges encountered by international students, with curriculum and instructional approaches representing the most significant concerns (Alavi and Mansor, 2011). These findings suggest that when Chinese international students reference intense emotional experiences without specifying valence, they may be predominantly recalling adaptation-related challenges rather than positive experiences. This interpretation is strongly supported by the remarkably high loading (0.878) of item *is6* on the Negative Intensity factor, with minimal cross-loadings on other dimensions.

Research literature demonstrates that when individuals reference heightened emotional awareness without specifying emotion types, this communication pattern predominantly indicates negative emotional experiences (Baumeister et al., 2001; Ekman and Cordaro, 2011). This occurs because negative emotions characteristically present with greater intensity and salience, producing more profound effects on individual psychological well-being. This association is particularly relevant in Chinese cultural contexts, which traditionally prioritize emotional restraint and effective self-regulation (Spencer-Rodgers et al., 2010). When Chinese students report heightened emotional awareness, this typically suggests they are experiencing emotional states that transcend normal regulatory parameters—a condition predominantly interpreted negatively within Chinese cultural frameworks (Spencer-Rodgers et al., 2010).

From a measurement perspective, item *is6* likely serves as an evaluation indicator for emotional regulation challenges or adaptation difficulties rather than positive cultural engagement. This reinforces that while the statement presents with linguistic neutrality, its interpretation within cross-cultural adaptation research frameworks demonstrates significantly stronger alignment with negative emotional intensity expressions.

Conversely, items “*is2*: “My body reacts very strongly to emotional situations while studying abroad” (在海外留学期间, 我的身体对情绪化的情境反应非常强烈。) and “*is3*: I have strong emotions while studying abroad. (在海外留学期间, 我的情感非常强烈)” demonstrated strong loadings (0.836 and 0.855 respectively) on the Positive Intensity (PI) factor, with minimal cross-loadings on other dimensions. This statistical pattern suggests a systematic tendency in how these items are interpreted by respondents.

One plausible explanation for this loading pattern may be found in how Chinese international students conceptualize situation-specific emotional reactions within cross-cultural contexts. Research has demonstrated significant connections between students’ personal development and cross-cultural competence acquisition (Gu et al., 2010), suggesting that emotional responses in specific cross-cultural situations can be framed as developmental opportunities rather than threats. Further support for this interpretation comes from studies characterizing cross-cultural experiences as simultaneously ‘rich and fragmented’ (Kim, 2017), where students navigate complex emotional terrain that includes both challenges and growth opportunities. Within this theoretical framework, the strong positive intensity loadings of situation-specific emotional items could reflect the tendency to interpret contextualized emotional reactions as part of the positive developmental process of cross-cultural adaptation.

The differential loading patterns between items with situational context (i*s2, is3*) and those without (*is6*) illuminates how similar emotional phenomena can represent either growth opportunities or adaptation challenges depending on their framing. The differential loading patterns of similarly worded items (*is2, is3, is6*) illuminates the complex duality of cross-cultural emotional experiences. While items *is2* and *is3* describing strong emotional reactions within specific situational contexts loaded strongly on Positive Intensity, an item describing general emotional intensity without contextual framing (item *is6*) loaded on Negative Intensity. This pattern suggests that contextual framing significantly influences how emotional intensity is interpreted in cross-cultural settings. When emotional reactions are anchored to specific situations, they appear more likely to be associated with positive developmental experiences, whereas generalized emotional intensity may evoke associations with adaptation challenges. The factor structure thus appears to capture the psychological complexity of navigating cross-cultural emotional experiences. Through engagement with diverse educational environments and cultural settings, international students often develop new perspectives and capabilities that facilitate navigation between host and home countries. Chinese international students at Malaysian universities may interpret situation-specific emotional intensities within this developmental framework.

Finally, during the pilot phase, Rasch analysis provided valuable item-level insights about the BEQ items within our Chinese international student sample. While Rasch modeling offers advantages in measurement quality and item diagnostics, it has limitations in discovering dimensions beyond those initially specified. Building on these pilot findings, our main study employed a sequential strategy: first conducting confirmatory factor analysis (CFA) of the original model, then exploratory factor analysis (EFA) when model fit proved poor and finally validating the new structure with a second CFA. The initial results showed the original BEQ structure inadequately represented emotional expressivity patterns in our sample. The subsequent analyses yielded a four-factor solution that better reflected the target population’s emotional expressivity. Validation of this revised structure through a follow-up CFA provided further evidence for its psychometric robustness and contextual appropriateness. By integrating Rasch modeling with a CFA–EFA–CFA analytic sequence, this study advances a methodologically rigorous and culturally attuned approach to the adaptation of psychological instruments. The identification of alternative factor structures highlights the significance of cultural context in shaping the dimensionality of emotional expressivity, suggesting meaningful divergence from the conceptualizations underlying the BEQ’s original development in Western populations.

## 6 Conclusion

Our findings reveal significant cultural nuances in emotional expressivity among Chinese populations, with important implications for cross-cultural measurement. The observed factor cross-loading pattern between Negative Expressivity (NE) and Positive Expressivity (PE) dimensions reflects the conceptual ambiguity surrounding emotional concealment in contemporary Chinese society. This psychometric finding stems from a cultural transition where traditional values framing emotional restraint as mature coexist with evolving perspectives influenced by globalization (Chen and Chen, 2010). For modern Chinese individuals in cross-culture context, particularly younger generations, emotional concealment occupies a complex interpretive position—simultaneously viewed as both a traditional virtue reflecting social competence and potentially a barrier to authentic self-expression (Soto et al., 2011).

The four-dimensional structure identified in our analysis provides empirical support for the conceptual distinction between emotional expression and intensity while highlighting valence-specific processing patterns. This refined model offers a more nuanced framework for understanding emotional experience and expression in Chinese populations. From a measurement perspective, these findings suggest specific adaptations to the BEQ model when applied in Chinese cultural contexts, including the adoption of a four-dimensional structure and modification of problematic items (specifically items *ne1*, *ne6*, and *pe1*) to enhance construct validity. Rather than indicating deficiencies in the original instrument, these structural modifications represent important advances in cultural adaptation research that contribute significantly to the integration of cross-cultural perspectives into developmental research (Nielsen and Haun, 2016).

Methodologically, this study makes several notable contributions to cross-cultural measurement adaptation. First, our approach demonstrates the value of combining rigorous factor analysis with culturally informed interpretation, revealing how statistical patterns (such as cross-loadings and factor migrations) can provide meaningful insights into cultural differences in emotional conceptualization. Second, we illustrate how apparent measurement ‘anomalies’ can be reframed as valuable cultural data points rather than statistical noise, offering a more nuanced alternative to the common practice of simply eliminating problematic items. Third, our identification of valence-specific emotional intensity dimensions provides a methodological template for examining how other seemingly unitary psychological constructs might manifest differently across cultural contexts. Finally, by documenting the specific process of adapting the BEQ for Chinese international students, we offer practical guidance for researchers undertaking similar cross-cultural adaptations of Western-developed psychological instruments.

When interpreting these results, several methodological limitations should be acknowledged. Our study utilized convenience sampling from just one public Malaysian university, potentially restricting the generalizability of our results to wider Chinese international student populations or Chinese individuals in other settings. Malaysia’s distinct cultural environment may foster adaptation experiences that differ significantly from those of Chinese students studying in Western nations or other Asian contexts. Furthermore, our convenience sampling approach may have introduced sampling biases affecting the representativeness of our findings. To strengthen and verify the structural adaptations we identified, future studies should employ more diverse sampling strategies encompassing various institutions and geographical areas. Although Rasch modeling was employed in the pilot phase to evaluate item-level functioning of the BEQ, examining item difficulty estimates and fit statistics, we did not conduct differential item functioning (DIF) analysis due to sample size constraints. This limitation means potential item bias across subgroups could not be fully evaluated. Despite this, the Rasch analysis informed subsequent validation by identifying potentially problematic items. Future research with larger, more diverse samples should incorporate DIF analysis to strengthen the instrument’s cross-cultural validity.

## 7 Implications

The substantial measurement discrepancies observed demonstrate how cultural context shapes the interpretation of emotional behavior, highlighting the critical importance of culturally appropriate assessment methods. These results emphasize meaningful cultural distinctions in the valuation and interpretation of emotional expression, indicating that assessment tools originating in Western frameworks require thoughtful adaptation when applied to Chinese students in international educational contexts (Olson et al., 2019). For researchers and practitioners working with Chinese populations, our findings necessitate a recalibration of assessment approaches to obtain more accurate measurements of emotional functioning. Future research should extend this investigation to include diverse geographic regions while exploring relationships between emotional expressivity and other psychological constructs in educational settings. Although this study employed a rigorous, multi-step analytic strategy (Rasch–CFA–EFA–CFA), the findings remain sensitive to sample size limitations. In particular, factor structural variations identified in the EFA phase may reflect both cultural context and methodological characteristics. Therefore, interpretations of newly extracted dimensions should be approached with caution, as over-attribution to cultural factors may risk overlooking measurement-related or analytical artifacts (Byrne and Van de Vijver, 2010).

These findings offer practical value for professionals working with Chinese international students in Malaysian educational settings. For educators in international contexts, our four-dimensional model provides a more accurate framework for understanding Chinese students’ emotional expressions, preventing misinterpretation of emotional restraint as disengagement or disinterest. Mental health professionals can employ this culturally adapted approach when assessing adjustment difficulties or psychological well-being, while cross-cultural trainers can better prepare students by explicitly addressing differences in emotional expression across cultures. We recommend pilot testing of our adapted model before implementation in new contexts or with diverse Chinese subgroups, as regional and individual differences may influence emotional expression patterns. This validation step ensures culturally appropriate assessment while avoiding inappropriate generalization of our findings.

## Data Availability

The raw data supporting the conclusions of this article will be made available by the authors, without undue reservation.
